# The Influenza Again

**Published:** 1892-02

**Authors:** 


### THE INFLUENZA AGAIN.

This winter witnesses the third annual appearance of the "Grippe" in this country. In the North it began about two months ago, and in a short time it had reached nearly every section of the Union. The arrival of the "Grippe" in this country after our people had enjoyed an immunity from the epidemic for a period of eleven years, was treated lightly at first, and people spoke of it with a careless unconcern that might have been used with reference to attacks of chicken-pox. But toward the end of its first season complications, principally pneumonia and an obstinate bronchitis, began to carry off a patient here and there, usually the debilitated and infirm, and it was realized then that the "Grippe" was not such an insignificant thing after all. Since that time it has claimed many victims.

The epidemic of one year ago was not so severe, and the present seems to be milder still. In fact, in Atlanta there have not been a great many cases of genuine influenza. To be sure, during the last two months there has been a sort of epidemic of colds, but certainly all of the cases which have received the lay or professional diagnosis of the "Grippe" were not really such.

But we wish to speak particularly of the treatment. In the three visits which the "Grippe" has made to us many have been the treatments followed, and nearly every physician has pursued his favorite method, which he found to answer the purpose exceedingly well.

In the New York *Medical Journal*, January 9th, Dr. J. Harrison Mettler, of Chicago, outlines the course of treatment which he usually pursues, and as we have had a satisfactory experience with practically the same methods we give the following brief description:

In the first place, absolute mental and physical rest is all important. A hot bath should be taken at night before retiring, after which the patient should get in bed between heavy blankets. Patient should take eight or ten grains of quinine, and a pill containing extract of opium (gr. ¼ to ½), camphor and ammonium carbonate (aa gr. ij). Instead of the latter pill we have usually used a ten-grain Dover's powder. A hot whiskey punch will be an advantage also. Thus pain is relieved, diaphoresis is established and sleep induced. In the morning the patient will feel much better. During this day he should remain in a warm room, and at intervals of two or three hours take some stimulus, as milk punch or brandy. If fever be present quinine should be given, two grains every three or four hours If fever persists and shows a tendency to rise toward evening, phenacetine may be employed. For the nervous exhaustion often seen the author uses the hypophosphites, or a pill of arsenic, strychnine, quinine and the dried sulphate of iron. We have depended principally on the use of phenacetine, four grains about every three hours. At night the patient may repeat the process of the night before. Diet should be light and nutritious, with milk as the staple article of food.

In the ordinary cases as they present themselves the above represents, we believe, an excellent method of management. No mention is made in the article under consideration of the coughs which are sometimes troublesome. For these we know of nothing better than a mixture containing equal parts of syrup of wild cherry, paregoric and Wyeth's compound syrup of white pine.

